# Size selection and placement of pedicle screws using robot-assisted versus fluoroscopy-guided techniques for thoracolumbar fractures: possible implications for the screw loosening rate

**DOI:** 10.1186/s12893-022-01814-6

**Published:** 2022-10-22

**Authors:** Sheng-yang Du, Jun Dai, Zhen-tao Zhou, Bing-chen Shan, Feng-xian Jiang, Jing-yan Yang, Lei Cao, Xiao-zhong Zhou

**Affiliations:** 1 Department of Orthopaedics, Second Affiliated Hospital of Soochow University, 1055 Sanxiang Road, Suzhou, Jiangsu China; 2 Department of Orthopaedics, First People’s Hospital of Xuzhou, 269 Daxue Road, Xuzhou, Jiangsu China; 3 Institute for Social and Economic Research and Policy, Graduate School of Arts and Sciences, Columbia University, New York, NY USA

**Keywords:** Thoracolumbar fracture, Robot-assisted surgery, Minimally invasive surgery, Screw loosening

## Abstract

**Background:**

There has been increased development of robotic technologies for the accuracy of percutaneous pedicle screw placement. However, it remains unclear whether the robot really optimize the selection of screw sizes and enhance screw stability. The purpose of this study is to compare the sizes (diameter and length), placement accuracy and the loosening rate of pedicle screws using robotic-assisted versus conventional fluoroscopy approaches for thoracolumbar fractures.

**Methods:**

A retrospective cohort study was conducted to evaluate 70 consecutive patients [34 cases of robot-assisted percutaneous pedicle screw fixation (RAF) and 36 of conventional fluoroscopy-guided percutaneous pedicle screw fixation (FGF)]. Demographics, clinical characteristics, and radiological features were recorded. Pedicle screw length, diameter, and pedicle screw placement accuracy were assessed. The patients’ sagittal kyphosis Cobb angles (KCA), anterior vertebral height ratios (VHA), and screw loosening rate were evaluated by radiographic data 1 year after surgery.

**Results:**

There was no significant difference in the mean computed tomography (CT) Hounsfield unit (HU) values, operation duration, or length of hospital stay between the groups. Compared with the FGF group, the RAF group had a lower fluoroscopy frequency [14 (12–18) vs. 21 (16–25), *P* < 0.001] and a higher “grade A + B” pedicle screw placement rate (96.5% vs. 89.4%, *P* < 0.05). The mean screw diameter was 6.04 ± 0.55 mm in the RAF group and 5.78 ± 0.50 mm in the FGF group (*P* < 0.001). The mean screw length was 50.45 ± 4.37 mm in the RAF group and 48.63 ± 3.86 mm in the FGF group (*P* < 0.001). The correction loss of the KCA and VHR of the RAF group was less than that of the FGT group at the 1-year follow-up [(3.8 ± 1.8° vs. 4.9 ± 4.2°) and (5.5 ± 4.9% vs. 6.4 ± 5.7%)], and screw loosening occurred in 2 out of 34 patients (5.9%) in the RAF group, and 6 out of 36 patients (16.7%) in the FGF group, but there were no significant differences (*P* > 0.05).

**Conclusion:**

Compared with the fluoroscopy-guided technique, robotic-assisted spine surgery decreased radiation exposure and optimizes screw trajectories and dimensions intraoperatively. Although not statistically significant, the loosening rate of the RAF group was lower that of than the FGT group.

## Background

Thoracolumbar fractures account for 30–60% of spinal fractures and are commonly seen in high-energy trauma patients [1, 2]. Burst fractures (AO spine types A3 and A4) represent the majority of thoracolumbar fractures [3]. Timely diagnosis and surgical stabilization are essential to prevent further neurological deficits and to reconstruct the sagittal balance, which can help avoid long-term complications [4–6]. Percutaneous pedicle screw fixation is the most commonly performed surgical procedure for the treatment of thoracolumbar spine fractures without neurological deficits.

In general, misplacement and inappropriate choice of pedicle screws negatively affect the internal fixation stability [7, 8]. Inserting the longer and larger screw accurately optimizes fixation strength [8, 9]. However, the benefits of larger screws must be weighed against the risks of pedicle breach or neurovascular injury [10, 11]. Therefore, the key to this technique lies in the determination of an appropriate-sized screw for maximum fixation strength while simultaneously respecting the structural integrity of the vertebral pedicles.

Some studies have reported that robot-assisted techniques provide more precise screw placement [12–14], and more recent literature shows that robotic assistance allows for the placement of screws with greater screw diameter and length compared with surgical navigation alone in minimally invasive lumbar fusion procedures [15]. However, to the best of our knowledge, few studies have compared the dimension selection and loosening rate of pedicle screws between robot-assisted techniques and fluoroscopy-guided percutaneous techniques for thoracolumbar fractures. In the present study, we compared radiological and clinical parameters from the immediate postoperative time to the 1-year follow-up between patients who underwent robot-assisted and conventional fluoroscopy surgical procedures.

## Materials and methods

### Patient selection

This was a retrospective cohort study. A consecutive sample of 70 patients with A3 and A4 thoracolumbar fractures from May 2018 to October 2020 was enrolled. Of the 70 patients reviewed, 34 and 36 were treated with robotic-assisted percutaneous pedicle screw fixation and fluoroscopy-guided percutaneous pedicle screw fixation, respectively, based on the following inclusion criteria: (1) thoracolumbar fracture classified as A3 or A4; (2) short-segment fixation with two additional screws at the single fracture vertebra; (3) no other fractures or organ injuries; and (4) absence of neurological deficits. The exclusion criteria were as follows: (1) incomplete clinical information; (2) combined anterior-posterior surgeries; (3) revision surgeries; (4) fracture displacement in the vertebral pedicle; and (5) structural spinal deformity, spinal tumour, or infection. Written informed consent was obtained from all patients. The present study was reviewed and approved by the Ethics Committee and Institutional Review Board of the Second Affiliated Hospital of Soochow University.

## Surgical techniques

### RAF group

**Fig. 1:**
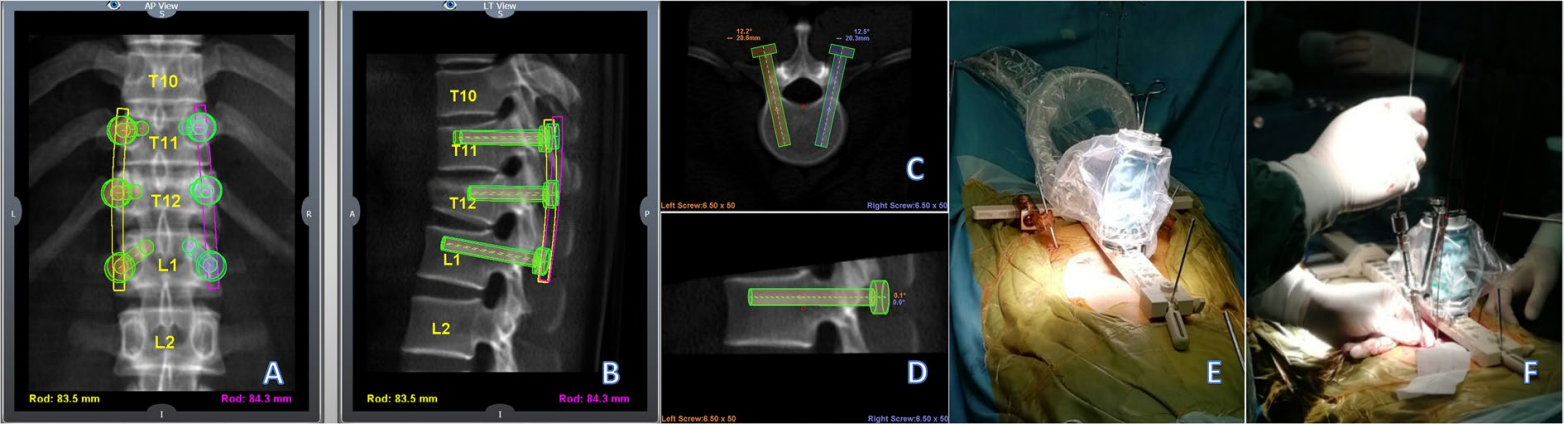
Perfect trajectories of the vertebrae based on the surgeon’s requests were planned (**A**–**D**). The manipulator was placed on the bone-mounted platform, and the appropriate pedicle screws were inserted (**E**, **F**)

Patients were placed in the prone position after general anaesthesia. The CT scan of the surgical region was uploaded to the workstation (Mazor Robotics Company, Israel) before surgery, and the perfect trajectories of the vertebrae based on the surgeon’s requests were planned (Fig. 1A–D). After the anteroposterior and oblique plane images were captured by the C-arm, the fluoroscopic images were automatically merged with the preoperative CT. A small (400 g, 9 cm tall, 5 cm diameter), parallel, 6-DOF robotic manipulator was then placed on the bone-mounted platform and aligned with a planned trajectory according to the surgeon’s instructions (Fig. 1E). After the paths of the screws were tapped with a thread tap through the dilated channels, the screws were inserted using handover guide wires (Fig. 1F). Rods were placed percutaneously from either the cephalad or caudal side with the assistance of screw extenders.

## FGF group

Patients were placed in the prone position after general anaesthesia. The fractured vertebrae were confirmed by fluoroscopy. Six transpedicular puncture needles were inserted through six small incisions using fluoroscopy, after which guide wires were inserted. The appropriate pedicle screws were then placed through the guide wires. The position of each screw was examined by fluoroscopy, followed by the insertion of titanium rods with the assistance of screw extenders.

## Data collection

**Fig. 2:**
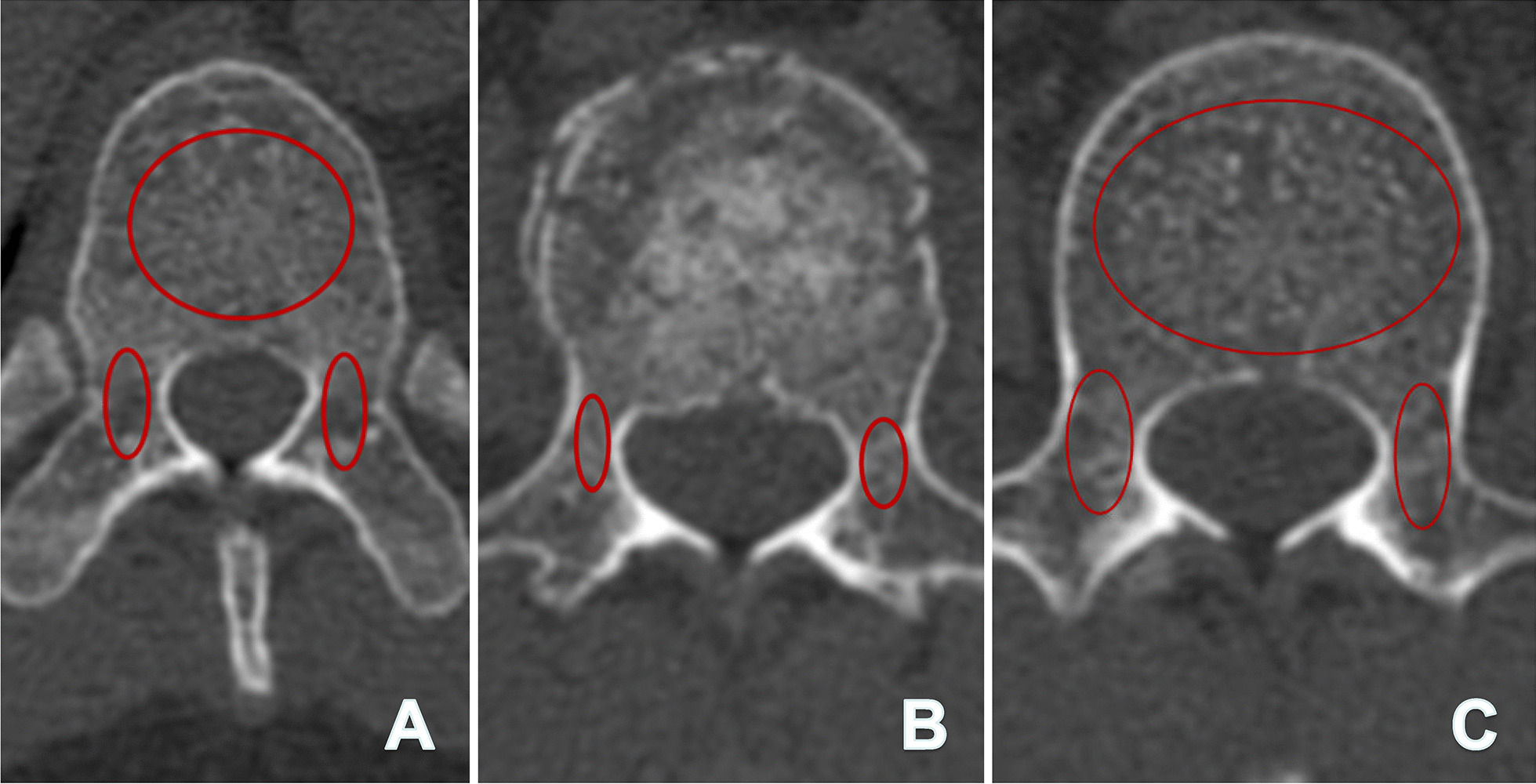
Measurement methods for mean CT Hounsfield Unit (HU) values: a 41-year-old male patient was diagnosed with a vertebral fracture in L1. **A** The mean CT Hounsfield Unit (HU) values of the T12 vertebral body (VB) were 176, and the right and left pedicles (excluding the cortical bone) were 133 and 136 respectively; **B** the mean HU values of the right and left pedicles of the L1 (fractured vertebra) were 157 and 144 respectively; **C** the mean HU values of the L2 VB was 164, and the right and left pedicle were 160 and 147 respectively. The mean HU values of the patient was 152.1 = [(176 + 133 + 136 + 157 + 144 + 164 + 160 + 147)/8]

Patient demographics and clinical characteristics were recorded. The mean CT Hounsfield Unit (HU) values were obtained from the measurement of preoperative CT measurements (Fig. 2). The duration of the operation, number of radiation cycles, amount of intraoperative blood loss, length of hospital stay, and cost were compared between the two groups. The surgical data obtained included the levels of instrument, and pedicle screw length, and diameter. Screw dimensions were obtained from the patient’s electronic health records, to which all operative implant records were uploaded. Considering that short-length pedicle screws were used for the fractured vertebral body, only screws in the vertebra above and below the fractured vertebral body were counted.

## Radiographic measurements

Two spine surgeons with more than 10 years of experience completed the measurements independently. The average value of the measured data was the final data. All data were measured using radiology software (Neusoft PACS/RIS) to reduce variability.

**Fig. 3:**
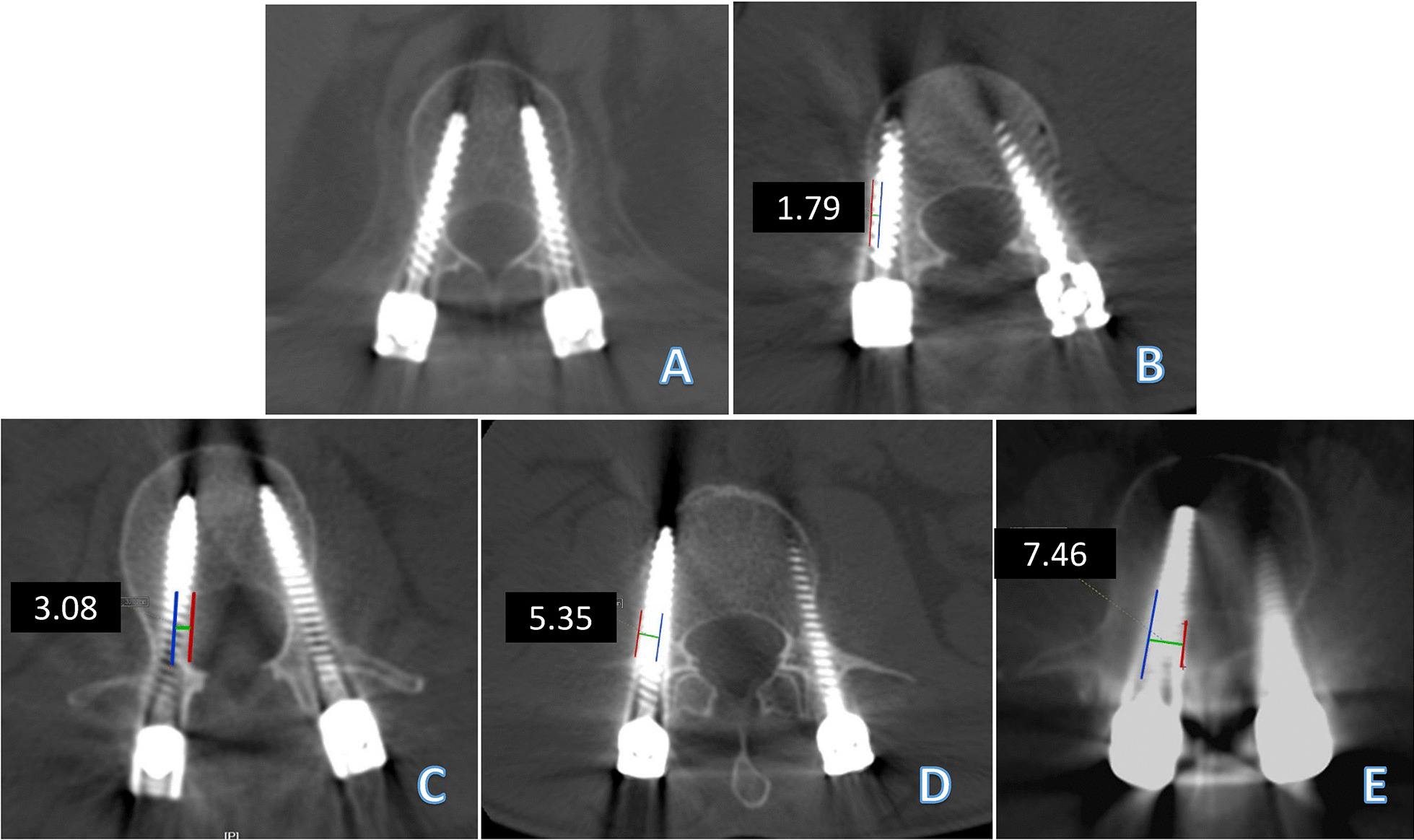
Grading used for pedicle perforation on the axial CT scan and the representative images: **A** both pedicle screws are completely within the pedicle (Grade A); **B** Grade B (< 2 mm) perforation of the lateral wall of the right pedicle and Grade A of the left pedicle; **C** Grade C (2–4 mm) perforation of the medial wall of the left pedicle and Grade A perforation of the right pedicle; **D** Grade D (> 4 mm) perforation of the lateral wall of both pedicles; **E** Grade E (> 6 mm) perforation of the medial wall of the right pedicle. The blue lines represent the medial or lateral wall of the vertebral pedicles; the red lines represent the edge of the pedicle screws; and the green lines represent the distance of the perforation

Based on the Gertzbein and Robbins scale [16], the accuracy of the pedicle screw placement was evaluated on postoperative CT images as follows (Fig. 3A–E): Grade A: screw completely within bone; Grade B: cortical breach of < 2 mm; Grade C: cortical breach of ≥ 2 mm and < 4 mm; Grade D: cortical breach of ≥ 4 mm and < 6 mm; and Grade E: cortical breach of ≥ 6 mm.

**Fig. 4:**
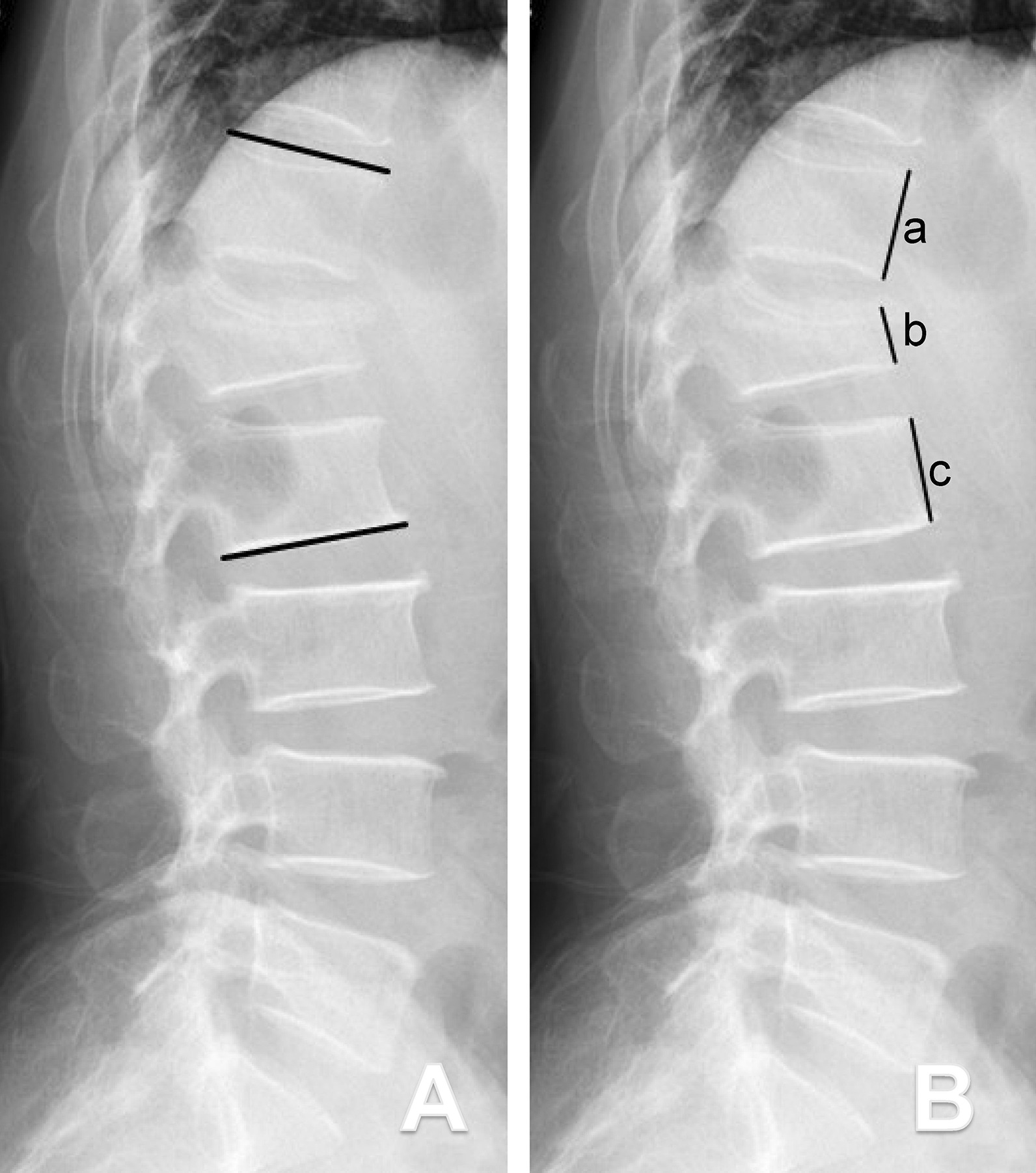
Schematic drawings showing the measurement methods for the sagittal kyphosis Cobb angles (KCA) and anterior vertebral height ratios (VHR). **A** The KCA was defined as the angle between the superior endplate of the upper vertebra(superior black line) and the inferior endplate of the lower vertebra (inferior black line) in accordance with Cobb’s method; **B** VHR = 2b/(a + c)

The pre- and postoperative sagittal kyphosis Cobb angles (defined by the upper endplate of the first vertebra above the fractured vertebra and by the lower endplate of the first vertebra below the fractured vertebra) and anterior vertebral height ratios (defined by the percentage of the mean values for the adjacent vertebrae) of the fractured vertebra were measured using radiographic images (Fig. 4).

**Fig. 5:**
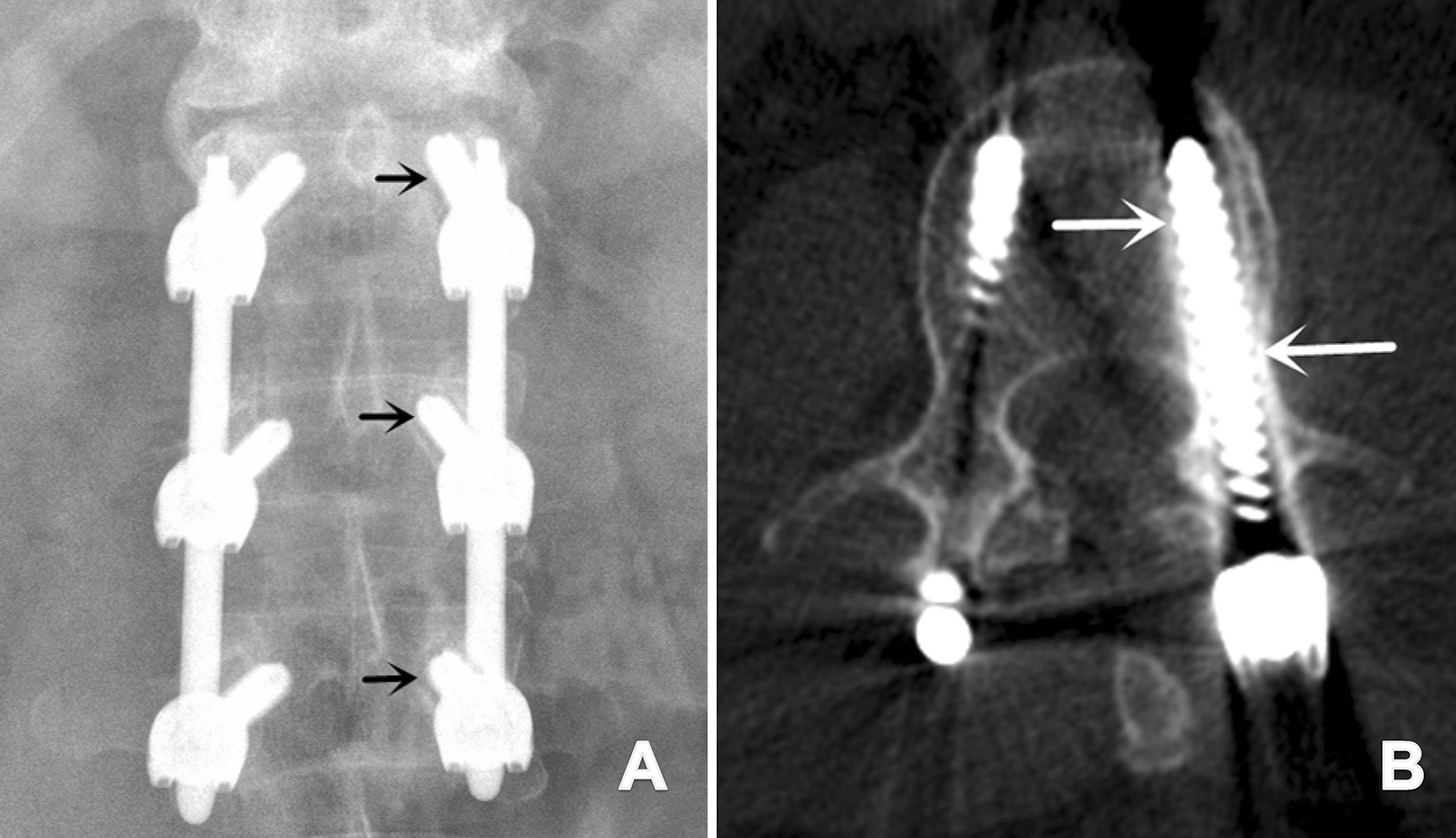
**A** Postoperative 1-year plain X-ray demonstrated left screw loosening at T12, L1, and L2 in a 39-year-old male who underwent short-segment fixation from T12 to L2 (black arrow: double halo sign); **B** postoperative 1-year CT demonstrated left screw loosening at L1 in a 47-year-old male who underwent short-segment fixation from T11 to L12 (white arrow: lucent zone)

Pedicle screw loosening was defined as a “double-halo sign” on plain X-ray or a “lucent zone” around on CT images, which were obtained 1 year postoperatively (Fig. 5) [17, 18].

### Statistical analysis

Descriptive statistics are presented as the mean ± standard deviation for normally distributed continuous variables; the median and interquartile range was used for nonnormally distributed continuous variables and the count (frequency) was used for categorical variables. Comparisons of baseline characteristics, which were stratified by group, were performed using Student’s *t* tests for normally distributed continuous variables, Wilcoxon rank-sum tests for variables with skewed distributions, and chi-square tests for categorical data. A p value < 0.05 was considered statistically significant. All analyses were performed using SPSS for Mac (IBM SPSS Statistics 23.0, SPSS Inc., Chicago, IL, USA).

## Results

Overall, 70 patients who underwent short-segment fixation were included in the study. Patient demographics and clinical characteristics were compared between the RAF and FGF groups. There were no significant differences between the groups in terms of age, sex, BMI, mean HU values, mechanism of injury, fracture level and type, and TLICS score. The baseline data of the two groups were comparable (Table 1).

**Table 1:** Baseline characteristics and perioperative parameters of all patients

Characteristic	RAF	FGF	Statistic (χ^2^/t)	*P* value
No. of pts	34	36	–	–
Mean age (years)	42.8 ± 8.7	39.8 ± 10.7	1.277	0.206
Sex			0.370	0.558
Male	25	26	–	–
Female	9	10	–	–
Mean BMI (kg/m^2^)	23.8 ± 2.6	24.0 ± 2.7	0.699	0.718
Mean HU values	184.4 ± 66.5	202.4 ± 80.4	− 1.026	0.311
Mechanism of injury			0.475	0.830
Traffic accident	22	22		
Fall	12	14		
Fracture level			0.826	0.972
T10	0	0		
T11	3	4		
T12	8	8		
L1	18	16		
L2	5	8		
Fracture type			0.214	0.543
A3	25	21		
A4	9	15		
TLICS score	4.5 ± 0.5	4.6 ± 0.5	− 0.448	0.656

*RAF *robot-assisted percutaneous pedicle screw fixation, *FGF *fluoroscopy-guided percutaneous pedicle screw fixation, *BMI *body mass index, *HU *Hounsfeld Unit, *TLICS Score *Thoracolumbar Injury Classification and Severity Score

## Clinical evaluation

The RAF group had significantly fewer radiation cycles (*P* < 0.001) but higher hospitalization costs (*P* = 0.001) than the FGF group (Table 2). No significant difference was observed between the two groups in terms of surgical time, intraoperative blood loss, or length of hospital stay (Table 2).

**Table 2:** Summary of operation values

Operative factors	RAF	FGF	Statistic (t/*Z*)	*P* value
Mean operation time (min)	114.1 ± 34.4	104.0 ± 18.1	1.552	0.125
Fluoroscopy frequency (times)	16.1 ± 3.1	22.6 ± 3.9	− 7.815	0.000^**^
Mean hospital stay (days)	7.5 ± 2.4	8.2 ± 2.5	− 1.279	0.204
Mean blood loss (mL)	93.8 ± 24.9	102.2 ± 27.8	− 0.963	0.339
Hospitalization expenses (RMB)	70529.6 ± 17224.5	58803.1 ± 9753.4	3.53	0.001^**^

*RAF *robot-assisted percutaneous pedicle screw fixation, *FGF *fluoroscopy-guided percutaneous pedicle screw fixation, *RMB *Renminbi

**P < 0.01, statistically significant

## Pedicle screw dimensions

There were 136 screws placed in the RAF group and 144 placed in the FGF group (screws in the vertebra above and below the fractured vertebra). Screws placed in the RAF group had both a larger screw diameter (RAF 6.04 ± 0.55 mm [range 5.0–7.0 mm]; FGF 5.78 ± 0.50 mm [range 5.0–6.5 mm]) and screw length (RAF 50.45 ± 4.37 mm [range 40–55 mm]; FGF 48.63 ± 3.86 mm [range 40–55 mm]) than screws placed in the FGF group (*P* < 0.001) (Table 3).

**Table 3:** Pedicle screw diameter and length

Variable	RAF	FGF	Statistic (χ^2^)	*P* value
No. of screws				
Total	136	144	5.309	0.379
T10	2	6		
T11	22	20		
T12	36	34		
L1	32	38		
L2	34	28		
L3	10	18		
Mean screw diameter (range), mm				
Total	6.04 (5.0–7.0)	5.78 (5.0–6.5)	− 3.067	0.000**
T10	5.5 (5.5)	5.17 (5.0–5.5)	–	–
T11	6.09 (5.5–7.0)	5.90 (5.5–6.5)	-0.975	0.330
T12	6.11 (5.5–7.0)	6.04 (5.5–6.5)	-0.332	0.740
L1	5.81 (5.0–6.5)	5.29 (5.0–6.0)	-4.281	0.000**
L2	6.12 (5.5–7.0)	6.07 (5.5–6.5)	-0.238	0.812
L3	6.20 (6.0–6.5)	5.94 (5.5–6.5)	-2.143	0.032*
Mean screw length (range), mm				
Total	50.45 (40–55)	48.63 (40–55)	-3.561	0.000**
T10	50 (50)	48.54 (45–50)	–	–
T11	50.00 (40–55)	49.50 (40–55)	− 0.320	0.749
T12	49.19 (45–55)	47.35 (45–55)	2.064	0.039*
L1	50.65 (40–55)	48.96 (40–55)	− 1.966	0.049*
L2	51.18 (45–55)	49.34 (45–55)	− 1.391	0.164
L3	53.00 (50–55)	48.33 (45–55)	− 3.144	0.020*

*RAF *robot-assisted percutaneous pedicle screw fixation, *FGF *fluoroscopy-guided percutaneous pedicle screw fixation

*P < 0.05; **P < 0.01, statistically significant

## Radiographic evaluation

In the RAF group, 91.2% of the 204 screws were well placed (grade A); the remaining screws were graded B (5.4%), C (2.9%), or D (1.0%), but no screw was graded E. In the FGF group, 80.1% of the 216 screws were graded A; the remaining screws were graded B (9.3%), C (6.9%), D (2.8%), or E (0.9%). The RAF group had a significantly higher ratio of clinically acceptable screws (grades A and B, 96.6%) than the FGF group (89.4%) (*P* < 0.05) (Table 4).

**Table 4:** Pedicle screw accuracy rate

Variable	RAF	FGF	Statistic (χ^2^)	*P* value
No. of screws	204	216		
Screw accuracy rate			11.750	0.019^*^
Grade A	186 (91.2%)	173 (80.1%)		
Grade B	11 (5.4%)	20 (9.3%)		
Grade A + B	197 (96.6%)	193 (89.4%)		
Grade C	5 (2.4%)	15 (6.9%)		
Grade D	2 (1.0%)	6 (2.8%)		
Grade E	0	2 (0.9%)		
Grade C + D + E	7 (3.4%)	23 (10.6%)		

*RAF *robot-assisted percutaneous pedicle screw fixation, *FGF *fluoroscopy-guided percutaneous pedicle screw fixation

*P < 0.05 statistically significant

The preoperative kyphosis Cobb angle (KCA) and anterior height ratio of fractured vertebra (VHR) were similar between the two groups. After surgery, the KCA was significantly corrected, and the VHR was well improved in both groups. The correction loss of the KCA (3.8 ± 1.8°) was less than that of the FGT group( 4.9 ± 4.2°), and the VHR of the RAF group (5.5 ± 4.9% ) was less than that of the FGT group (6.4 ± 5.7%) at the 1-year follow-up, but there were no significant differences (Table 5).

**Table 5:** Summary of radiographic measurements

Variable	RAF	FGF	Statistic (χ^2^/Z/t)	*P* value
No. of pts	34	36	–	–
Kyphosis Cobb angle (KCA) (°)				
Preoperative KCA	20.2 ± 5.8	20.9 ± 4.3	− 0.613	0.542
Postoperative KCA	8.2 ± 4.8	8.7 ± 3.9	− 0.494	0.621
Postoperative KCA correction	12.0 ± 4.4	12.2 ± 3.4	− 0.256	0.798
KCA at 1-y FU	11.9 ± 4.7	− 9.3 ± 3.9	− 1.374	0.174
Correction loss at 1-y FU	3.8 ± 1.8	4.9 ± 4.2	− 1.485	0.138
Anterior height ratio of fracture vertebral (VHR) (%)				
Preoperative VHR	62.0 ± 11.2	60.7 ± 14.3	0.419	0.676
Postoperative VHR	84.6 ± 9.9	82.5 ± 11.4	0.812	0.420
Postoperative VHR Correction	22.6 ± 12.1	21.9 ± 12.9	− 0.106	0.910
VHR at 1-y FU	79.2 ± 7.9	76.1 ± 10.7	1.356	0.180
Correction loss at 1-y FU	5.5 ± 4.9	6.4 ± 5.7	− 0.975	0.329
Screw loosening rate of pts (%)	2 (5.9%)	6 (16.7%)	2.009	0.156

*RAF *robot-assisted percutaneous pedicle screw fixation, *FGF *fluoroscopy-guided percutaneous pedicle screw fixation, *KCA *Kyphosis Cobb angle, *AVHR *anterior vertebral height ratios, *1-y FU *1-year follow-up, *pts *patient

Screw loosening occurred in 2 out of 34 patients (5.9%) in the RAF group, and 6 out of 36 patients (16.7%) in the FGF group. There was no statistically significant difference in terms of the screw loosening rate between the two groups (Table 5).

## Discussion

Our study had several main findings as follows. First, the robot-assisted technique provides more precise screw placement in minimally invasive spine surgery than conventional fluoroscopy. Second, robot-assisted techniques allow for the placement of screws with greater screw diameter and length compared with fluoroscopy-guided techniques. That is, robotic assistance may allow for safe placement of the “optimal screw”. Third, we found that robot-assisted technology can reduce intraoperative radiation exposure and increase treatment costs. Finally, although there was no significant difference, there was a lower correction loss at the 1-year follow-up and the loosening rate of the screw in the RAF group.

In recent years, there have been many studies on the robot’s advantage in accuracy and selection of pedicle screws, offering benefits for both patients and surgeons [15, 19–21]. Preoperative CT is used to plan screw trajectories, and intraoperative fluoroscopy is applied to register images. The robot then guides the surgeon to the appropriate trajectory, with precise screw placement, less redirection [22, 23], and a lower axial trajectory [7]. Molliqaj et al. [12] reported a clinically acceptable screw placement accuracy under robotic guidance of up to 93.4%. Karim et al. [15]found that surgeons, with the assistance of a robot, tended to choose larger-diameter and longer screws in the minimally invasive surgery-transforaminal lumbar interbody fusion when compared with intraoperative navigation alone, particularly when using sizes that they may have previously thought to be too large. Our results are somewhat similar to these studies. In our study, compared with fluoroscopy-guided techniques, robotic assistance helped surgeons to select the larger screws and ensured accuracy in the screw placement.

Theoretically, these benefits may reduce the screw loosening rate, maintain the fractured vertebral height, and provide a more stable environment for the healing of the thoracolumbar fracture. Numerous in vitro experiments have demonstrated that perfect central-axis pedicle screws without redirection are significantly more powerful in terms of screw loosening force and axial pull-out strength and that larger-diameter and longer screws have greater resistance to screw pull-out [22, 24–26]. However, these studies have inherent limitations associated with cadaveric studies and/or bone models, with which it is difficult to mimic the actual clinical situation. A previous study showed that the instability of pedicle screw increased the loosening rate and loss of correction in vertebral fractures [27]. Ohba et al. investigated the association between the pull-out length and screw loosening 1 year after surgery, and found 81.8% of patients with loosened pedicle screws had developed the screw pull-out phenomenon postoperatively [7]. Therefore, to determine whether these “optimal” screws may provide a more stable environment, our study compared the kyphosis Cobb angle of fractured segments, the anterior height ratio of fractured vertebra, and the screw loosening rate between the two groups at the 1-year follow-up.

Our study revealed that the loosening rate of the RAF group was lower than that of the FGT group (5.9% vs. 16.7%), and the correction loss of the KCA (3.8 ± 1.8° vs. 4.9 ± 4.2°) and VHR (5.5 ± 4.9% vs. 6.4 ± 5.7%) of the RAF group was less than that of the FGT group at the 1-year follow-up, although there were no significant differences in the groups. Of note, 5 out of 8 patients with loosened pedicle screws (62.5%) in this study had showed the obvious screw pull-out phenomenon (data not shown). Three possible interpretations were considered to explain why the significant differences were not seen in our study. First, the mechanisms that contribute to pedicle screw loosening are certain to be multifactorial, in addition to the screw placement and sizes mentioned, including high forces of spinal rods, bone mineral density (BMD), postoperative physiological movements, and paraspinal muscle function [28–30]. Previous studies have shown that BMD plays an important role in the stability of screws [31, 32], so BMD was assessed by the CT HU values to make the two groups comparable in our study. The patients’ CT HU values were relatively high in the two groups, because most of these patients were young, which might increase the holding power of screws and avoid loosening. Second, the sample size of fracture cases was relatively small, and all patients were recruited from a single centre. Last, the 1-year follow-up period was short, and most of patients required removal of the internal fixation after fracture healing. Overall, although the advantages of robotic surgery in the terms of screw stability were not confirmed in our study, the robotic technology is one possible way to insert pedicle screws following the trajectory, which has been designed to increase purchase in the bone and obtain adequate fixation, especially in osteoporotic bone.

Moreover, we also found that robot-assisted technology helped to reduce intraoperative radiation exposure, which is consistent with previous studies [33]. Another clinically relevant finding is that the treatment cost in the RAF group was higher than that in the conventional group, which was due to the additional costs of disposable materials.

## Limitations

Our study has several limitations. First, the sample size of fracture cases was relatively small, and all patients were recruited from a single centre. Future studies with larger sample sizes are needed to replicate the findings. Second, data on the trajectory angles, the radians and forces of spinal rods, and misaligned spinal rods, all of which can cause screw loosening, were not available. Finally, further experiments are necessary to design an adequate trajectory, which can significantly increase the stability of the pedicle screw.

## Conclusion

Compared with fluoroscopy-guided techniques, robotic assistance can help a surgeon to optimize screw trajectories and dimensions intraoperatively. Although not statistically significant, these findings are a possible way to increase bone purchase and reduce the screw loosening rate for thoracolumbar fractures. In addition, with increased experience and proficiency in robot-assisted techniques, surgeons should be more confident in their screw selection, especially when using sizes that they may have previously considered too large.

## Data Availability

The datasets used and/or analysed during the current study are available from the corresponding author on reasonable request.

## Abbreviations

RAF: Robot-assisted percutaneous pedicle screw fixation
FGF: Fluoroscopy-guided percutaneous pedicle screw fixation
KCA: Sagittal kyphosis Cobb angles
VHA: Anterior vertebral height ratios
CT: Computed tomography
HU: Hounsfield unit
BMI: Body mass index
BMD: Bone mineral density
TLICS Score: Thoracolumbar Injury Classification and Severity Score
1-y FU: 1-year follow-up
pts: Patient
